# A 3D-Printed Sole Design Bioinspired by Cat Paw Pad and Triply Periodic Minimal Surface for Improving Paratrooper Landing Protection

**DOI:** 10.3390/polym14163270

**Published:** 2022-08-11

**Authors:** Yilin Xiao, Dayong Hu, Zhiqiang Zhang, Baoqing Pei, Xueqing Wu, Peng Lin

**Affiliations:** 1 Department of Aircraft Airworthiness Engineering, School of Transportation Science and Engineering, Beihang University, Beijing 100191, China; 2 Aircraft/Engine Integrated System Safety Beijing Key Laboratory, Beijing 100191, China; 3 Department of Biomedical Engineering, College of Engineering, Shantou University, Shantou 515063, China; 4 School of Biological Science and Medical Engineering, Beihang University, Beijing 100191, China

**Keywords:** TPMS, bionic cushion, 3D-printed sole, energy dissipation, landing protection

## Abstract

Paratroopers are highly susceptible to lower extremity impact injuries during landing. To reduce the ground reaction force (GRF), inspired by the cat paw pad and triply periodic minimal surface (TPMS), a novel type of bionic cushion sole for paratrooper boots was designed and fabricated by additive manufacturing. A shear thickening fluid (STF) was used to mimic the unique adipose tissue with viscoelastic behavior found in cat paw pads, which is formed by a dermal layer encompassing a subcutaneous layer and acts as the primary energy dissipation mechanism for attenuating ground impact. Based on uniaxial compression tests using four typical types of cubic TPMS specimens, TPMSs with Gyroid and Diamond topologies were chosen to fill the midsole. The quasi-static and dynamic mechanical behaviors of the bionic sole were investigated by quasi-static compression tests and drop hammer tests, respectively. Then, drop landing tests at heights of 40 cm and 80 cm were performed on five kinds of soles to assess the cushioning capacity and compare them with standard paratrooper boots and sports shoes. The results showed that sports shoes had the highest cushioning capacity at a height of 40 cm, whereas at a height of 80 cm, the sole with a 1.5 mm thick Gyroid configuration and STF filling could reduce the maximum peak GRF by 15.5% when compared to standard paratrooper boots. The present work has implications for the design of novel bioinspired soles for reducing impact force.

## 1. Introduction

Human lower extremities are highly susceptible to injuries in various sports, such as running, basketball, and tennis. It is considered that lower extremity injuries are partly attributable to the impact force caused by jumping and landing activities during sports [1]. For example, running can generate a vertical GRF of 2.5~3 body weight (BW) and joint reaction forces of 3.6~4.2 BW in the knee and 10 BW in the hip, while for moderate-impact jumping and higher-impact jumping, the GRF might be up to 4.6 BW and 11.6 BW, respectively. These impact forces are exacerbated in paratrooper landing. To avoid radar and anti-aircraft weapon systems, paratroopers need to jump at an altitude of about 300 m or even lower [2]. Due to the low jump height combined with a very short cushioning time, paratroopers are subjected to huge ground impacts when landing, resulting in an extremely severe risk of injury. As a result, they have always been the personnel at the highest risk of injury in the military [3,4], and their lower extremity injuries account for the highest proportion of parachuting injuries [5]. Consequently, lower extremity protective equipment for paratroopers has attracted extensive attention and has considerable application prospects.

Actually, the human body can also attenuate the ground impact through a series of movements during landing [6]. Kovács et al. [7] suggested that a forefoot landing strategy could result in greater energy dissipation in the lower extremity musculature. According to the study by Blackburn et al. [8], the forward inclination of the trunk during landing will increase the maximum flexion angles of the hips and knees and decrease the peak GRF as well as the activity level of the quadriceps femoris. However, the effectiveness of these cushioning mechanisms is limited by biomechanical or morphological constraints, and it is very difficult to modify them to improve the cushioning capability during landing [9]. Therefore, when subjected to very high impact, it is very necessary for humans to reduce the impact with the help of auxiliary instruments. Shoes, as the most direct medium of human contact with the ground, can provide a cushioning capability to protect the human body from ground impact injuries by attenuating the vertical GRF [10] and altering the rate of loading on lower extremities [11]. In particular, this cushioning capability becomes even more crucial during landings from unexpected drops [12]. Accordingly, it is of great significance to improve the shoe cushioning capability for human body protection. Laurent et al. [13] found that soles made of soft materials could attenuate the impact on the human body by comparing a large number of running data. Dols et al. [14] compared barefoot and five different shoes and found that shoes with air chambers could maximize the ground contact time. The famous Vaporfly running shoe designed by Nike uses an airbag construction and a carbon fiber plate to provide excellent cushioning and a significant increase in energy return [15,16]. Lam et al. [17] found that shoes with a soft midsole have better rearfoot cushioning performance and perceived comfort but poorer forefoot cushioning performance as compared with hard shoes. A reasonable explanation is that the sole experienced “bottom out” during landing due to the relatively thin forefoot and soft midsole [17,18,19], thus leading to a very high GRF. Similar research was also carried out by Panagiotis [20]. He changed the hardness of the sole by 3D printing a midsole structure with different relative densities to reduce the peak GRF. Ali et al. [21] explored the significance of a variable-dimension helical spring (VDS) in the shoe midsole to improve the stiffness, energy absorption, and energy return. Their results revealed that the VDS midsole had a higher force-bearing capacity and better capability to attenuate the impact force than a midsole with a uniform-dimension helical spring. Given the higher thickness of the midsole compared to the insole and outsole, midsoles play an important role in the overall shock absorption of a shoe [9]. By considering the impact mitigation performance of different materials and the material arrangement within the soles corresponding to areas of high loading, it was concluded that the midsole can be even more important in the design of high-energy-absorbing shoes [22]. Alycia et al. [23] investigated the impact attenuation properties of jazz shoes, and they thought that equal emphasis should be given to the cushioning capability of the rearfoot and forefoot. Ramanathan et al. [24] investigated the effect of sole thickness on the cushioning performance, and the results showed that shoes with thicker soles imposed a detrimental effect on maintaining the stability of the ankle-subtalar joint complex. From the literature review, previous studies have suggested that it is very important to choose the suitable sole stiffness and thickness for improving the cushioning performance of the sole, thus providing better protection to the human body.

Most of the cushioned shoes mentioned above were designed for daily sports, such as running or basketball, with good shock attenuation performance in running and jumping. However, few soles were designed for paratrooper landing or high-height drops, in which the human body is subjected to a higher risk of injuries. Therefore, it has become highly urgent to design a new sole structure to improve the cushioning performance during landing or dropping from high heights while reducing the risk of injury.

To address this issue, in the present work, based on our previous work on the impact resistance biomechanism of the cat [25,26,27,28,29,30], a bioinspired cushioning sole utilizing STF and TPMS was designed and fabricated by additive manufacturing. STF was used to mimic the adipose tissue found in cat paw pads, which is formed by a subcutaneous layer enclosed within a dermal layer and exhibits typical viscoelastic behavior. To mimic the geometric shape of cat paw pads, several holes were arranged in the rearfoot and forefoot of the sole and filled with STF. The TPMS lattice structure was used to fill the midsole. The bionic sole was analyzed using FE models, mechanically tested using quasi-static compression, drop hammer, and drop landing tests, and then compared to standard paratrooper boots and sports shoes.

## 2. Specimen Design and Fabrication

**Figure 1:**
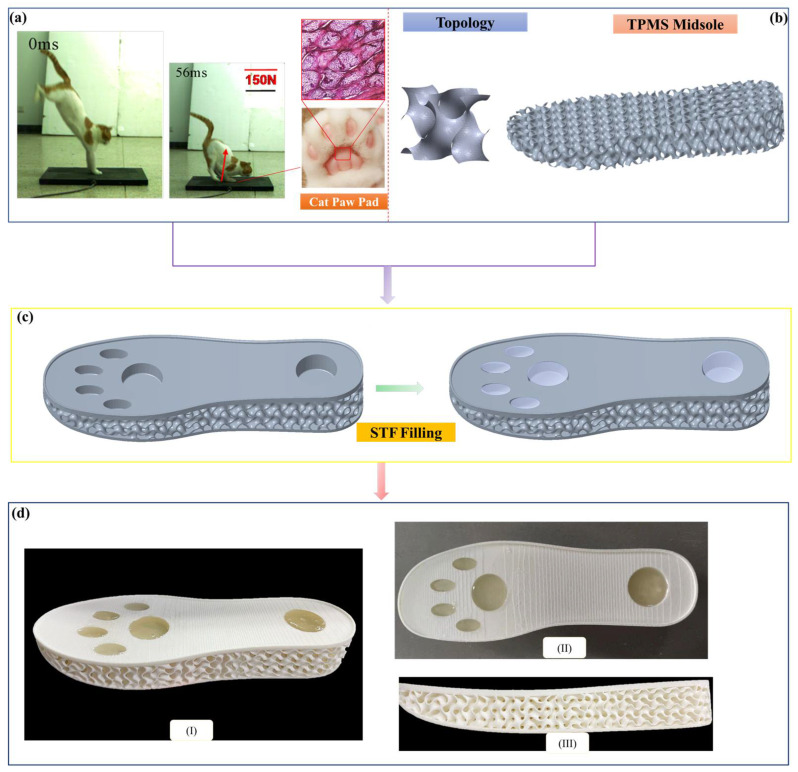
A bionic cushion sole design inspired by cat paw pads and TPMS: (**a**) image and microstructure of cat paw pads; (**b**) schematic diagram of TPMS midsole; (**c**) schematic diagram of sole with cat paw pad-shaped holes and TPMS midsole filled with STF; (**d**) cushion sole with different view orientations fabricated by additive manufacturing.

Figure 1 presents the schematic diagram of the cushion sole with the cat paw pad and TPMS configurations. The profile of the sole is based on the sole of standard paratrooper boots, the size of which is 275.51 mm (0.90 inch) in length and 93.64 mm (0.31 inch) at the maximum width. This would be approximately size 8/8.5/42 for UK/US/EUR. The maximum thickness of the sole is 37 mm (0.12 inch).

### 2.1. Mimicking Cat Paw Pads

Cats are known for their excellent jumping ability, which was developed over a long period of natural evolution [25,26,27,28,29,30]. Even though they are subjected to huge impact forces, as high as many times their body weight, they can land gracefully without injury when impacting the ground, as shown in Figure 1a. Their paw pads are considered to play a key role in attenuating the GRF during landing because they are the only parts of the body in contact with the ground. To thoroughly investigate the impact resistance biomechanism of cat paw pads, we conducted macroscopic mechanical properties tests at various frequencies, microstructure observation using the section staining technique and micro-CT scan, and finite element analysis [25,26]. Based on histological and micro-CT images, the paw pads of cats were found to be a typical layered structure, as highlighted in Figure 1a, which can be approximately divided into three layers: the epidermal layer, dermal layer, and subcutaneous layer. Similar layered structures were also found in canine paw pads [31]. The epidermal layer, which is in direct contact with the ground during locomotion, is composed of the toughest material, allowing it to withstand the intense ground–pad interaction. The subcutaneous layer, which consists of adipose tissue, is the softest material. The dermal layer encompassing the subcutaneous layer can be considered a hydrostatic system, and its predominant energy dissipation mechanism is attenuating the ground impact and dissipating the impact energy [25,26,31]. In addition, the mechanical test results revealed that cat paw pads, as a typical biological material, exhibit nonlinear and viscoelastic behaviors, which can contribute to reducing the impact displacement as the impact velocity increases. The structural features and impact resistance mechanism of the cat paw pad provided bioinspiration for the cushion sole design to reduce paratrooper lower extremity injuries during landing.

The designed sole with the configuration of a cat paw pad is shown in Figure 1c,d, in which several holes are arranged in the rearfoot and forefoot to mimic the shape of a cat paw pad. The TPMS porous structures were chosen as the matrix with stiffening characteristics to mimic the porous frames in cat paws. The depth of each hole is equal to half the thickness of the sole at its location. STF was used to fill all of the holes, inspired by the adipose tissue in cat paw pads, which consists of a subcutaneous layer surrounded by a dermal layer. STF is a type of non-Newtonian fluid that is widely used in the fields of army armor, damping systems, and shock absorbers. Its viscosity increases as the shear strain rate increases, similar to the viscoelastic behavior of cat paw pads: when there are no forces applied, it behaves like a liquid, but it turns into a very stiff solid-like structure in the presence of high shear rates. This distinctive viscosity variation would be beneficial in attenuating vertical displacement when the impact velocity increases, and more impact energy can also be dissipated by STF, providing better protection for human lower extremities subjected to intense impact.

STF was fabricated by dispersing micro-sized silicon dioxide (SiO_2_) particles (25% wt.) in polyethylene glycol (PEG). To ensure a uniform distribution of SiO_2_ particles in the medium, an ultrasonic bath was utilized during the mixing procedure. SiO_2_ was gradually added to PEG and manually mixed. After reaching the gel stage, the mixture was immersed in an ultrasonic bath and then vacuum-sealed overnight to eliminate air bubbles. Finally, the holes were filled with STF, which was uniformly distributed within the holes.

### 2.2. TPMS Description and Fabrication

The lattice structure is a type of architecture material frequently found in nature and engineering, and it is characterized by a unit cell consisting of struts, plates, or surfaces and tessellated in various patterns to create a three-dimensional structure. Due to the porous characteristics of lattice structures, they have a good cushioning capability that allows for more flexibility in tailoring the response to impulse loads than traditional materials, making them suitable for shoe sole designs [32,33]. A very famous product with a lattice sole is the Futurecraft 4D shoe. Recently, TPMS has received extensive attention due to its excellent performance and light weight. Therefore, we used a lattice structure of TPMS in the midsole, as shown in Figure 1b.

TPMS is a surface created mathematically [34,35,36]. It locally minimizes the surface area for a given boundary; therefore, TPMS has no edges or corners, and the mean curvature at each point on the surface is zero. Four types of TPMS are Diamond, Gyroid, Primitive, and IWP, respectively, which can be described by the following equations:

(1) $$\mathrm{Diamond}:\;\sin\frac{2\pi x}{l}\times \sin\frac{2\pi y}{l}\times \sin\frac{2\pi z}{l}+\sin\frac{2\pi x}{l}\times \cos\frac{2\pi y}{l}\times \cos\frac{2\pi z}{l}+\cos\frac{2\pi x}{l}\times \cos\frac{2\pi y}{l}\times \sin\frac{2\pi z}{l}=0$$

(2) $$\mathrm{Gyroid}:\;\cos\frac{2\pi x}{l}\times \sin\frac{2\pi y}{l}+\cos\frac{2\pi y}{l}\times \sin\frac{2\pi z}{l}+\cos\frac{2\pi z}{l}\times \sin\frac{2\pi x}{l}=0$$

(3) $$\mathrm{Primitive}:\;\cos\frac{2\pi x}{l}+\cos\frac{2\pi y}{l}+\cos\frac{2\pi z}{l}=0$$

(4) $$\mathrm{IWP}:\;2\left(\cos\frac{2\pi x}{l}\times \cos\frac{2\pi y}{l}+\cos\frac{2\pi y}{l}\times \cos\frac{2\pi z}{l}+\cos\frac{2\pi z}{l}\times \cos\frac{2\pi x}{l}\right)-\left(\cos2x+\cos2y+\cos2z\right)=0$$

Using Equations (1)–(4), the commercial software Matlab was used to create the geometry model and output TPMS stereolithography (STL) files. Then, STL files were imported into the Geomagic CAD software to define the thickness of TPMS for generating solid architectures. Finally, holes were punched in the TPMS solid architectures, and the sole shape was cut by Boolean operations using the sole shape profile (see Figure 1c) for 3D printing.

A hyperelastic material called thermoplastic polyurethane (TPU) with excellent wear and chemical resistance properties [37] was utilized to fabricate the sole by Selective Laser Sintering (SLS) 3D printing technology. This material is widely used in footwear industries. An EP-500 3D printer (E-Plus-3D, Beijing, China) with a print accuracy of 0.3 mm and commercial TPU Luvosint X92A-2 sinter powder (Lehmann & Voss & Co., Hamburg, Germany) were used for 3D printing. The optimized 3D printing processing parameters are summarized as follows: print speed of 4 mm/s, preheated temperature of 60 °C, operating temperature of 98 °C, laser power of 45 W, laser scan speed of 7.6 m/s, laser scan spacing of 100 μm, powder feed temperature of 65 °C, and layer thickness of 0.2 mm. After processing, the samples were allowed to cool for about 1 h in the equipment chamber before being removed from the printer and then blasted by a spray gun to remove non-sintered powder particles.

**Figure 2:**
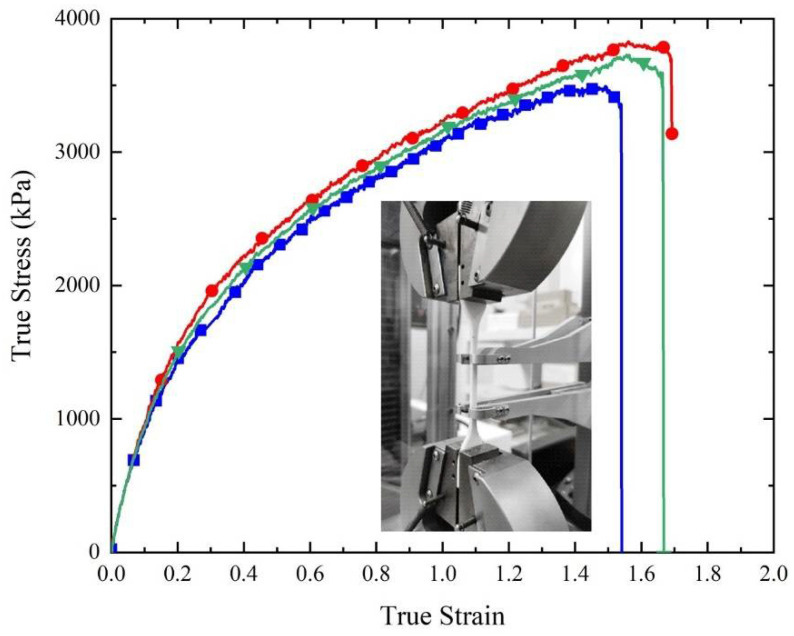
Stress–strain curves of three TPU tensile specimens.

**Figure 3:**
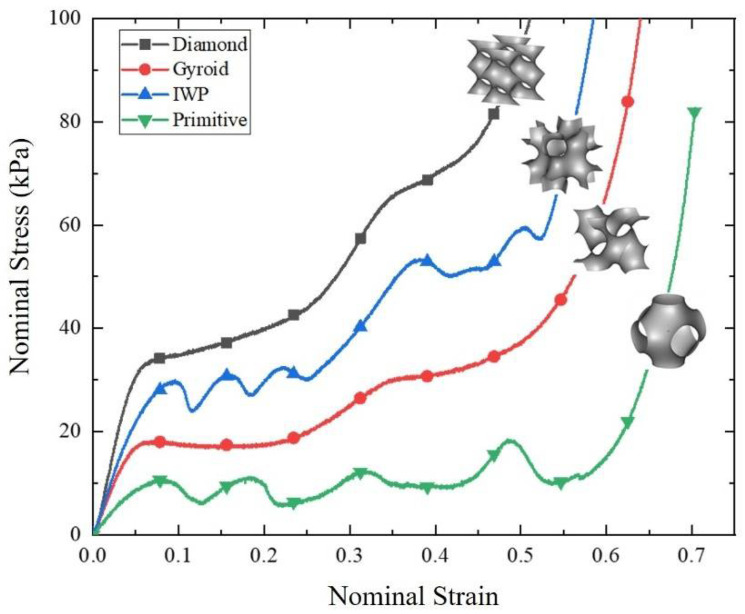
Nominal stress–nominal strain curves of four types of TPMS.

The stress–strain curve of TPU was measured according to ASTM D412, as shown in Figure 2, where the tensile specimen with a density of 1.20 g/cm^3^ is also presented. In the figure, the curves with different symbols represent different tensile tests. Cube-shaped TPMS specimens with four different topologies, as shown in Figures S1–S3, were printed with the dimensions: 60 mm × 60 mm × 60 mm in three directions, and quasi-static uniaxial compression tests were conducted to obtain nominal stress–strain curves and compare their mechanical properties. Figure 3 shows the nominal stress–strain curves of four different cubic TPMS specimens, and the corresponding deformation processes are presented in Figures S4–S7. All specimens were deformed in a progressive crushing process. In Figure 3, it can be observed that all of the curves exhibit a similar trend to the conventional honeycomb structure, characterized by three distinct regions: the linear elastic region, plateau region, and densification region. Diamond has the highest compression load and maximum stiffness, making it suitable for high-height landings that require more impact energy absorption and higher load capacity to avoid bottoming out. IWP has the second highest compression load, while Primitive has the lowest, but both exhibit dramatic load fluctuations, which is unfavorable for cushioning and makes them unsuitable for use as a shoe sole application. As compared to IWP, Gyroid has a relatively smooth compression load and a greater compaction strain, indicating a good energy absorption capacity and making it suitable as an energy-absorbing structure for shoe soles, despite having a lower compression load than IWP. Consequently, we selected TPMS with Diamond and Gyroid topologies to fill the midsole.

## 3. Experimental Methods

### 3.1. Quasi-Static Compression Tests

**Figure 4:**
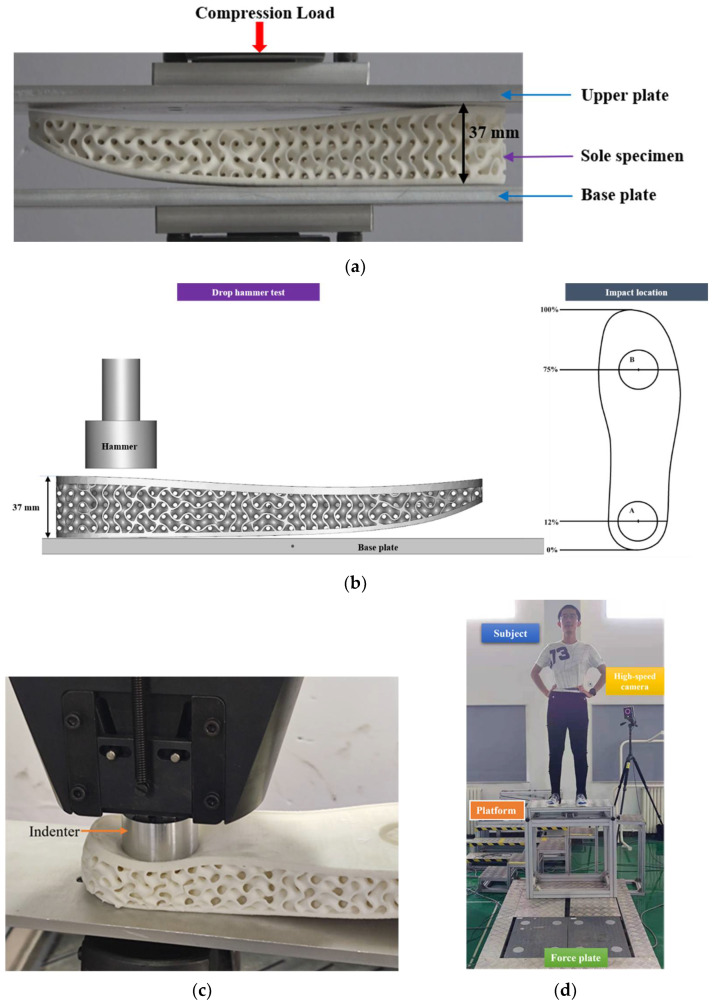
Experimental methods and facilities. (**a**) Quasi-static compression test; (**b**) drop hammer test; (**c**) quasi-static indenter compression test compared to drop hammer tests; (**d**) drop landing tests.

Quasi-static compression tests were performed using an Instron 5985 universal test machine under displacement control with a loading rate of 2 mm/min at room temperature to assess the quasi-static mechanical properties of the shoe soles that would be experienced by a sole during standing. The sole was sandwiched between two rectangular plates made of 6061 aluminum alloy with dimensions of 320 mm×120 mm×2 mm, as shown in Figure 4a, and these plates were clamped to the test machine. The load–displacement curves were measured with a sampling frequency of 1000 Hz using the test machine transducer, and the whole deformation process was recorded using a camera. When densification was reached, the tests were terminated.

### 3.2. Drop Hammer Tests

Dynamic impact tests were conducted using the Instron Ceast9350 drop hammer test machine to assess the impact attenuation properties of soles filled with STF, inspired by ASTM F1976 [38]. As illustrated in Figure 4b, the impact locations on the forefoot and rearfoot were adopted from ASTM F1976 at 12% and 75% of the top of the sole length, centered in the medio-lateral direction, respectively. The impact locations correspond to the positions of the STF-filled holes used to mimic the cat paw pad (see Figure 1d). The diameter of the drop hammer was 45 mm, and its mass was 9.1 kg. The height of the hammer could be adjusted to obtain different impact energy levels. The impact energy was varied from 15 J to 35 J with a 5 J interval at the rearfoot and 10 J to 20 J with a 5 J interval at the forefoot. The sole specimens were placed and attached to a rigid base plate with silicone to prevent sole slippage. The impact force history was measured by a load cell, which was located on the top of the hammer, with a sampling frequency of 5000 Hz.

In addition, quasi-static indenter compression tests, as shown in Figure 4c, were also performed with an indenter of the same size as the drop hammer and the same compression locations as dynamic impact tests (A and B in Figure 4b), and the results were compared with drop hammer test results to further highlight the dynamic enhancement effect of STF filling.

### 3.3. Drop Landing Tests

Drop landing tests were carried out to verify the cushioning performance of the designed cushion sole. Three subjects (mean (SD) age = 24.0 (1.3) years; height = 172.0 (4.5) cm; mass = 65 (5.2) kg) landed on a force plate (AMTI, Watertown, MA, USA) from heights of 40 and 80 cm, respectively, as shown in Figure 4d. None of the subjects had a history of previous surgery on the lower extremities, neurological or joint degenerative diseases, or vestibular or visual disturbances. The subjects were asked to learn the standard half-squat parachute landing (HSPL) technique, which includes flexing the lower extremities with the knees, ankles, and forefeet hugging each other and keeping the plantar parallel to the ground [39]. HSPL differs from the parachute landing fall (PLF) employed in most countries [4] and is called “three hugging and one parallel” in the training material of the China Airborne School [39]. The landing process was recorded by a high-speed camera (Photron, Tokyo, Japan) at 250 FPS. Vertical GRF data were measured by the force plate with a sampling frequency of 1000 Hz. All subjects were required to perform 10 landings consecutively at each height to obtain the average of the data, and then the force data were normalized to the body weight of the subject to reduce its contribution to variations among individuals [40]. The drop landing tests were conducted in accordance with the Declaration of Helsinki (2013) and approved by the Science and Ethics Committee of the School of Biological Science and Medical Engineering in Beihang University.

It should be noted that the soles were secured to the subject’s feet by adhesive tape during drop landing tests.

## 4. FE Model

**Figure 5:**
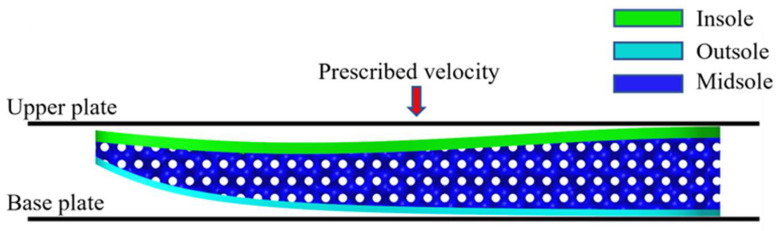
FE model of the sole with Gyroid configuration under quasi-static compression.

The FE models of Gyroid and Diamond TPMS soles without STF filling were built using the software ABAQUS to simulate quasi-static compression tests, respectively. Taking the FE model of the Gyroid sole as an example, as shown in Figure 5, the FE model mainly consisted of three parts: the upper plate, lower plate, and sole model. The upper and lower plates are defined as a rigid body. The sole model was a sandwich structure with an outsole, insole, and TPMS midsole. The midsole was connected to the outsole and insole through tied connections. The outsole and insole were meshed with 4-node tetrahedron elements, while the TPMS midsole was meshed using reduced-integration quadrilateral elements and triangular elements. The Yeoh hyperelastic constitutive model was used to describe the material mechanical properties of TPU, and the strain energy density functional *W* was defined as:

(5) $$W=C_{10}\left(I_{1}-3\right)+C_{20}{\left(I_{1}-3\right)}^{2}+C_{30}{\left(I_{1}-3\right)}^{3}$$

where $C_{ij}$ values are material constants and were obtained from the experimental curve in Figure 2 by the least-square fitting method.

In the FE model, the lower plate was fixed, while the upper plate moved downward with a prescribed velocity of 1 m/s [38] to simulate the quasi-static compression procedure. A surface-to-surface contact type was defined between the upper plate and insole and between the outsole and base plate, while penalty-based general contact was adopted to simulate the complex interaction between the insole, outsole, and midsole, including self-contact. The friction coefficient was set to 0.2 [38]. ABAQUS/EXPLICIT with an automatic time step was chosen as the solver, and the contact force exerted on the upper plate was selected as the output at a time interval of 0.001 s.

## 5. Results and Discussion

### 5.1. Results of Tests and FE Simulations under Quasi-Static Compression Conditions

**Figure 6:**
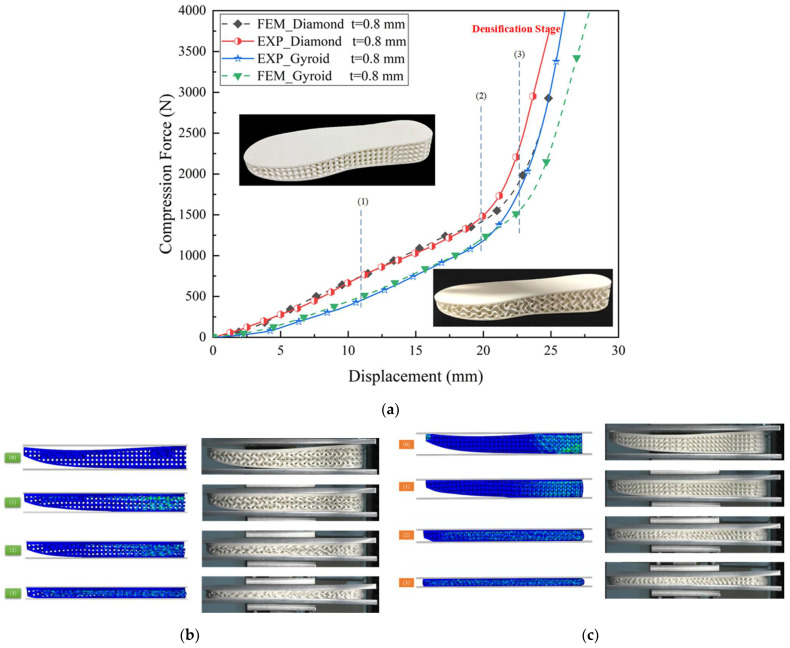
Comparison between compression test and FE results under quasi-static conditions for Gyroid and Diamond soles with a thickness of 1 mm. (**a**) Compression force versus displacement curves: (1) 11 mm; (2) 20 mm; (3) 23 mm; (**b**) compression deformation process of the Gyroid sole; (**c**) compression deformation process of the Diamond sole.

Soles with Gyroid and Diamond configurations were fabricated by SLS technology, and quasi-static compression tests were performed to obtain mechanical properties and compare them with numerical results. The thickness of the Gyroid and Diamond was 0.8 mm. Figure 6 shows the compression force–displacement curves and the corresponding deformation process obtained from FE simulations and compression tests for soles with different TPMS configurations under quasi-static compression conditions. It can be observed in Figure 6a that the compression force–displacement curves can be clearly divided into two stages: the force rising stage and densification stage, which has no plateau region and is quite different from the curves of cubic TPMS specimens (see Figure 3). The reason may be the increase in the contact area. As shown in Figure 6b,c, at the beginning of the compression tests, the insole and outsole were not in full contact with the upper plate and base plate, respectively. As the compression continued, the contact area grew larger and larger, resulting in an increasing compression load until it entered the densification stage due to self-contacting surfaces.

Figure 6 also presents a direct comparison between experimental and FE results. In Figure 6a, it can be seen that the compression force–displacement curves of soles with Gyroid and Diamond configurations exhibit a similar trend between tests and FE simulations. The numerical prediction could reproduce the global experimental phenomenon very well, and the compression deformation test and FE simulation results are in good agreement in Figure 6b,c. However, once the displacement exceeded 20 mm, the predicted compression loads were significantly smaller than the experimental ones, as shown in Figure 6a, indicating that the experimental results reached the compaction zone earlier than the FE results. The differences became more and more significant with the increase in displacement. This was mainly due to the following two reasons. First, the shell thickness was neglected in the contact analysis of FE models, which delayed the contact of the collapsed layers and resulted in an overestimation of the predicted densification displacement. Second, the semi-melted powder particles attached to the surface of the specimens increased the thickness of the TPMS midsole and reduced the densification displacement, although not contributing to mechanical stiffness and strength [41]. Considering the complexity of the actual structures, the accuracy of the numerical simulation was generally acceptable.

**Figure 7:**
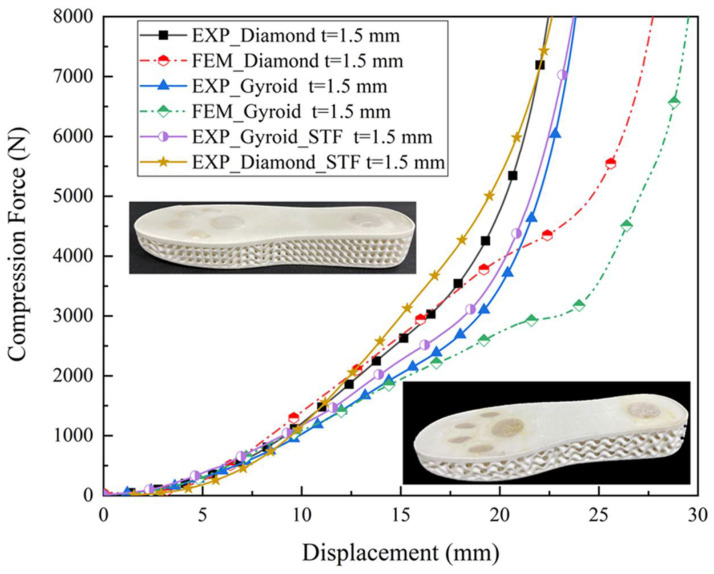
Compression force–displacement curves of Gyroid and Diamond soles of 1.5 mm thickness with and without STF filling.

To prevent lower extremities from being injured by the high load generated by the sole bottoming out, the compression force just before entering the densification stage was chosen as the maximum cushioning load of the sole. The maximum cushioning loads of Gyroid and Diamond soles with a thickness of 0.8 mm were 1447.7 N and 1798.5 N, respectively. Further, FE analysis was performed by varying the thickness from 0.8 to 1.5 mm with an interval of 0.1 mm, and the corresponding cushioning loads increased to 3086.6 N and 4270.0 N for Gyroid and Diamond soles, respectively. Figure 7 shows the quasi-static compression experimental results and the corresponding FE results for the 1.5 mm thick Gyroid and Diamond soles. Generally, the numerical prediction results are in good agreement with experimental results, although there are some discrepancies in the densification stage due to the effect of the contact definition in the FE model and semi-melted powder particles, as mentioned above. A thickness of more than 1.5 mm was not chosen because it had very limited compressible space, making it easy to enter densification, which is not beneficial for cushioning. Additionally, 1.5 mm thick Gyroid and Diamond soles with cat paw pad configurations filled with STF were also compressed and compared with the same soles without STF filling to investigate the influence of STF filling on the quasi-static mechanical behavior. The compression force–displacement curves for the Gyroid and Diamond soles with and without STF filling are presented in Figure 7, and the results show that the effect of STF filling was not considerable in general, and only a slight increase in load might be attributable to the confining pressure.

### 5.2. Drop Hammer Test Results

**Figure 8:**
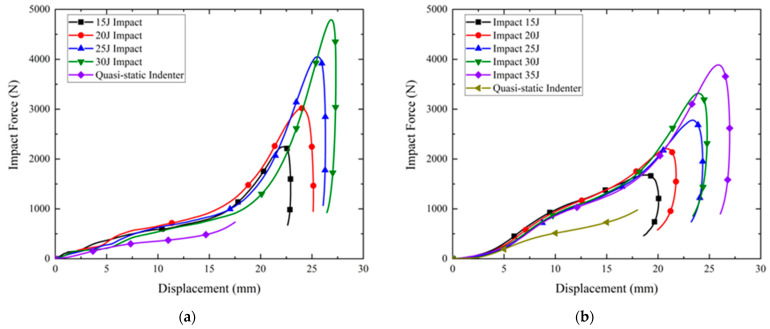
Results of drop hammer tests at rearfoot: (**a**) Gyroid; (**b**) Diamond.

Figure 8a,b present the results of drop hammer tests at different impact energies and quasi-static indenter compression tests at the rearfoot for Gyroid and Diamond soles with a thickness of 1.5 mm and STF filling, respectively. All of the curves of the drop hammer tests exhibit a similar trend, characterized by an increase in load followed by a sudden decrease. Here, it should be noted that the Gyroid sole appeared to bottom out at an impact energy of 30 J; the drop hammer test at an impact energy of 35 J was not conducted on this sole to avoid damage.

In Figure 8, clearly, both the Gyroid and Diamond soles show higher impact forces under impact conditions than under quasi-static conditions. This could be attributed to the inherent viscosity increase behavior of STF at high shear rates during the impact process. When the hammer struck the impact location (A in Figure 4b), the impact force caused local buckling deformation of TPMS, while a solidified STF formed and propagated along the impact direction due to a high impact velocity. The solidified STF consequently improved the structural rigidity and load-bearing capability, and a large amount of impact energy was dissipated through the deformation of the solidified STF during the impact. This dynamic enhancement effect could be affected by the impact velocity. However, in the drop hammer tests, as the velocity variation (from 1.8 to 2.8 m/s, corresponding to 15~35 J impact energy) was relatively limited, the difference between impact force–displacement curves at different impact energy levels was not significant, especially when the displacement was less than 20 mm before the densification stage. Furthermore, by comparing the impact force–displacement curves of Gyroid and Diamond soles at different impact energies, it was intuitively seen that at the same impact energy, the impact force level of the Diamond sole was higher than that of the Gyroid sole before entering the densification stage, indicating that the Diamond sole had higher stiffness and load-bearing capacity, which is very consistent with the quasi-static compression results (see Figure 6 and Figure 7). The Diamond sole also demonstrated superior energy absorption to the Gyroid sole, since the Gyroid sole entered the densification stage earlier than the Diamond sole at high energies of 25 J and 30 J.

**Figure 9:**
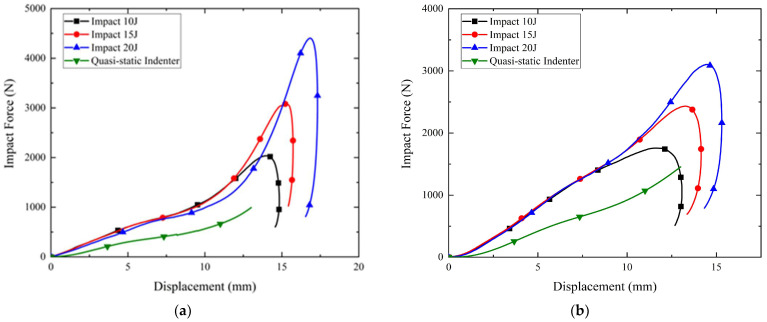
Results of drop hammer tests at forefoot: (**a**) Gyroid; (**b**) Diamond.

In Figure 9, similar phenomena can be observed in the drop hammer test results under impact energies of 10, 15, and 20 J and in the quasi-static indentation compression test results at the forefoot of the Gyroid and Diamond soles. Based on the above results, it can be concluded that STF filling can serve to improve the load-bearing and cushion capacities of the sole.

### 5.3. Drop Landing Test Results

In the drop landing tests, five types of shoes were used: Diamond and Gyroid soles with a thickness of 1.5 mm and filled with STF and the Gyroid sole with a thickness of 1.5 mm and no STF filling, with standard paratrooper boots and sports shoes (Under Armour, Baltimore, ML, USA) as a comparison. For convenience, they are named ES-A, ES-B, ES-C, BS-A, and BS-B, respectively. The thicknesses of ES-A, ES-B, ES-C, and BS-A were kept at 37 cm, while the thickness of BS-B was 35 cm.

**Figure 10:**
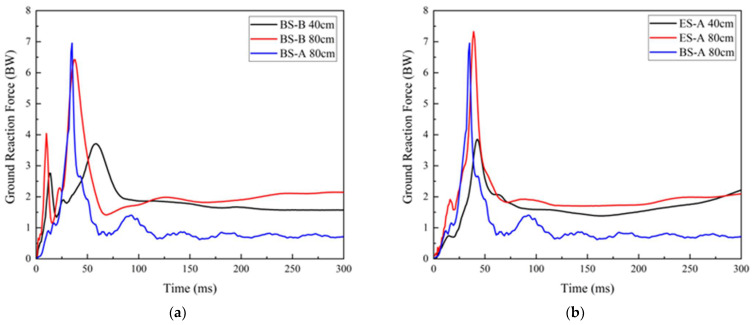

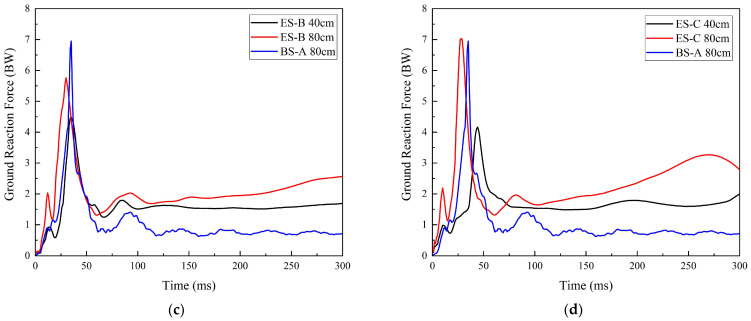
Representative ground reaction force curve of each working condition: (**a**) BS-B; (**b**) ES-A; (**c**) ES-B; (**d**) ES-C.

Figure 10 shows representative time history curves of measured vertical GRFs for ES-A, ES-B, ES-C, and BS-B dropped from heights of 40 cm and 80 cm, respectively. For BS-A, considering its actual use condition, only drop landing tests from a height of 80 cm were carried out, and the representative time history curve of GRF is also presented in Figure 10 for comparison. With the increase in drop height, the GRF increased significantly. As compared with 40 cm, subjects experienced a higher peak GRF at 80 cm, and the peak GRF appeared earlier. As a result, it can be concluded that an increase in landing height might increase the GRF experienced during landing and thus contribute to an aggravated risk of lower extremity injuries [4,42].

**Figure 11:**
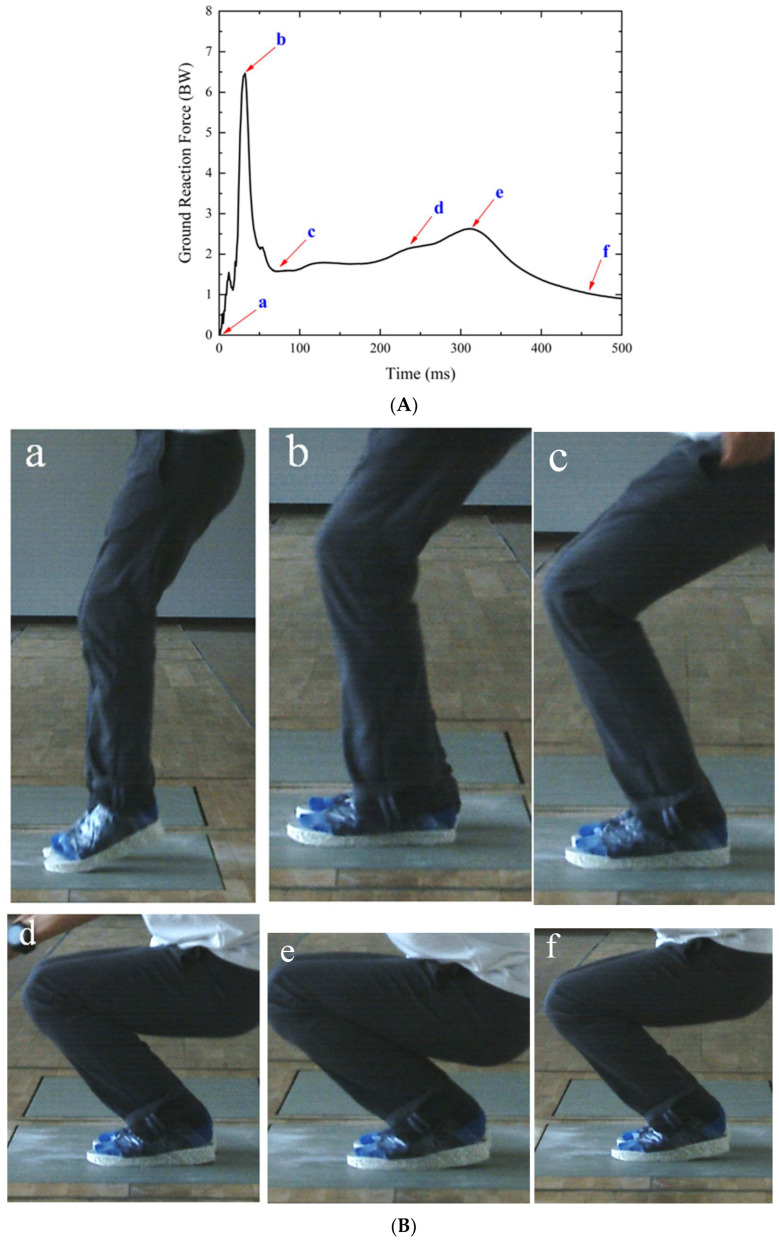
Results of drop landing tests: (**A**) representative GRF curve; (**B**) motion of lower extremities during landing.

As seen in Figure 10, all of the vertical GRF–time curves exhibit a similar tendency characterized by two peaks. These two peaks were also reported in Refs. [3,40,43,44,45] and are considered to be the peak vertical GRF at forefoot contact and heel contact, respectively. A typical GRF time history curve was selected and is presented in Figure 11A, and corresponding high-speed camera images of the drop landing process are also presented in Figure 11B. When the forefoot came into contact with the force plate (see a in Figure 11B), the GRF increased rapidly to the first peak (see a in Figure 11A); thereafter, GRF rose dramatically to the second peak (see b in Figure 11A) due to the sole compaction when the entire sole struck the force plate. Slight flexion of the lower extremities can also be observed, as reported by Decker et al. [45]. They thought that this lower extremity posture meant that the knee was more prepared to transfer energy to the larger and more proximal muscles, such as the hip extensors. Skinner et al. [46] considered that humans typically adopt this strategy by bending their knees to actively absorb much of the collision, possibly to avoid the discomfort of stiff-legged landings when landing on rigid ground. Furthermore, as shown by b to f in Figure 11a,b, after the second peak value, GRF decreased rapidly, followed by an almost constant fluctuation, and large rotations occurred in the joints of ankles, knees, and hips. The hip and knee joints are assumed to be major energy dissipation mechanisms throughout this process [42]. Based on the above results, it can be concluded that the effect of shoes on the biomechanics and energy dissipation mechanisms during drop landing are very complex [43]. We focused on the effect of sole cushioning properties on reducing the peak GRF.

**Figure 12:**
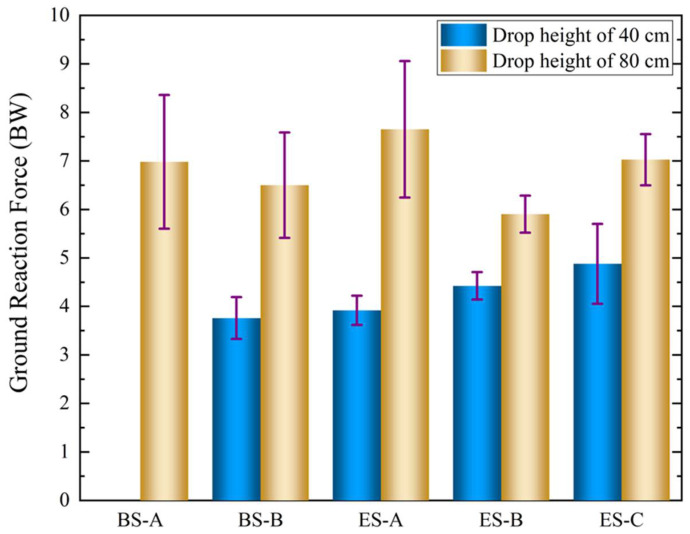
Peak vertical GRFs of different soles at different heights.

The peak vertical GRF is commonly used to quantify the amount of force exerted on the parachutist during landing [4], and it is also an essential risk factor for evaluating lower extremity injuries [42]. The mean and standard deviation of the peak vertical GRF for different soles obtained from drop landing tests are summarized in Table 1 and plotted in Figure 12. In drop landing tests from a height of 40 cm, BS-B had the lowest peak GRF, indicating that sports shoes had a better cushioning capacity at a low drop height, whereas soles with TPMS configurations (ES-A, ES-B, and ES-C) all had higher peak vertical GRFs than BS-B. The peak vertical GRFs of ES-B and ES-C with a thickness of 1.5 mm and STF filling were significantly higher than that of ES-A with a thickness of 1.5 mm and no STF filling, demonstrating the effect of STF filling on load-bearing capacity enhancement. As the height increased from 40 to 80 cm, the peak vertical GRF increased significantly. The peak vertical GRF of 6.98 ± 1.4 BW for the paratrooper boots BS-A was slightly higher than that of 6.2 ± 1.0 BW in Table 2 reported by Aziz et al. [4], which was obtained from PLF landing experiments with an average height of 54 cm from foot to mattress for amateur parachutists. This might be due to the effects of the drop height and landing posture. For PLF landing from drop heights of 93 cm and 137 cm in Table 2 [47], the peak vertical GRFs were 1.34 and 2.0 times higher than that of BS-A from a drop height of 80 cm, respectively. This demonstrates that the drop height has a significant effect on the GRF. Additionally, based on the results reported by Niu et al. [48] in Table 2, it can be observed that the peak vertical GRFs during HSPL landing experiments from drop heights of 32 cm, 52 cm, and 72 cm under the barefoot condition reached 16.95 ± 2.59 BW, 18.88 ± 3.46 BW, and 23.98 ± 4.21 BW, significantly higher than those when wearing BS-A, indicating that the sole plays a role in attenuating the GRF.

In Figure 12, it can also be observed that the peak vertical GRFs of five soles differed significantly. For ES-C, although it showed a good cushioning capacity at 40 cm, it had the highest peak at 80 cm, even surpassing BS-A, probably due to the fact that the “too soft” TPMS cushion structure of ES-A underwent densification (“bottom out”). ES-A failed to achieve the desired cushioning effect, and its peak vertical GRF was close to that of BS-A. The possible reason might be that ES-A has higher stiffness and strength, resulting in a higher GRF, and is not suitable for cushioning when drop landing from a height of 80 cm, although it perhaps has a greater cushioning potential at higher heights. In contrast, the values of the mean and standard deviation of the peak vertical GRF for ES-B were lower than those for the other soles. Meanwhile, the maximum value of the peak vertical GRF for ES-B was the lowest compared to those of the other four types of soles and was 15.5% lower than that of BS-A, showing that ES-B had the best cushioning capacity during drop landing from a height of 80 cm. Moreover, the weights of BS-A and ES-B were 1045.6 g and 514.4 g, respectively. It can be observed that ES-B was only about half the weight of BS-A due to the high specific strength and specific stiffness of the TPMS sole. In addition, ES-B was actually ES-C filled with STF, and their difference in the cushioning capacity was mainly due to STF filling, which reflects the fact that STF filling can improve the cushioning capacity.

Based on the above results, it can be concluded that ES-B (the Gyroid sole with a thickness of 1.5 mm and filled with STF) is the optimal choice for reducing the peak GRF during landing from a height of 80 cm, and it was subsequently processed into paratrooper boots, as shown in Figure S8.

## 6. Conclusions

In the present study, driven by the demand for reducing the peak vertical GRF and improving lower extremity injury protection for paratroopers during landings, a bionic cushioning sole inspired by TPMS and cat paw pads was designed and fabricated via the 3D printing technique. The main conclusions are as follows:

(a) The configuration of a cat paw pad was mimicked by arranging holes in the rearfoot and forefoot of the sole, and STF was used to fill all of the holes to mimic the hydrostatic system observed in cat paw pads, which is formed by a subcutaneous layer enclosed within a dermal layer and has typical nonlinear and viscoelastic characteristics. TPMSs with Gyroid and Diamond topologies were chosen for the midsole due to their stable load and high cushioning capacity demonstrated in quasi-static uniaxial compression tests of four typical types of cubic TPMS specimens. Then, the designed sole was fabricated with the SLS technique.

(b) The influence of TPMS thickness on the cushion load of soles with Gyroid and Diamond configurations was investigated using quasi-static compression tests and FE simulations. The numerical predictions of compression deformation were in good agreement with quasi-static compression experiments. The compression load versus displacement curves obtained from FE simulations also qualitatively and quantitatively matched well with experimental results before entering the compaction zone. As the thickness increased from 0.8 to 1.5 mm, the load increased from 1447.7 N to 3086.6 N for the Gyroid sole and 1798.5 N to 4270.0 N for the Diamond sole, respectively. Moreover, dynamic impact tests were performed using a drop hammer to assess the mechanical behavior of the soles with Gyroid and Diamond configurations and STF filling during impact, and the results were compared with those of quasi-static indenter compression tests. From the results, it was observed that both the Gyroid and Diamond soles showed higher impact force under impact conditions than under quasi-static conditions due to the inherent viscosity increase behavior of STF at high shear rates under an impact force.

(c) Five kinds of soles were tested in drop landing tests at heights of 40 cm and 80 cm to assess the sole cushioning performance. Experimental results indicated that standard sports shoes had the best cushioning capacity at a low drop height. However, at a height of 80 cm, the Gyroid sole with a thickness of 1.5 mm and STF filling provided the best cushioning capacity. It was able to reduce the maximum vertical GRF by 15.5% and weight by 50% compared to standard parachute boots. Moreover, the values of the mean and standard deviation of the peak vertical GRF of this sole were also lower than those of the other soles. Thus, the Gyroid sole with a thickness of 1.5 mm and STF filling was finally adopted as the sole of paratrooper boots to improve lower extremity injury protection.

The present work can provide a reference for utilizing bionic inspiration and TPMS for designing high-efficiency cushion footwear to reduce landing injuries in humans. Furthermore, future research will combine the developed soles with exoskeletons to prevent lower extremity injuries in paratroopers during landing, as shown in Figure S8. In addition, the durability of the design will be investigated by examining the cushioning capacity after realistic fatigue of the rear and fore parts of the developed soles.

## Figures and Tables

**Table 1:** Mean and standard deviation of peak vertical GRFs for five soles from drop landing tests at heights of 40 cm and 80 cm.

Sole Types	Peak GRF at 40 CM (BW)		Peak GRF at 80 CM (BW)		
	Mean	Standard Deviation	Mean	Standard Deviation	Improvement Relative to BS-A
BS-A			6.98	1.38	0
BS-B	3.76	0.43	6.50	1.09	6.9%
ES-A	3.92	0.3	7.65	1.41	−9.6%
ES-B	4.88	0.28	5.90	0.38	15.5%
ES-C	4.42	0.82	7.02	0.53	6.5%

**Table 2:** Peak vertical GRFs from parachute landing experiments.

Landing Technique	Shoe Type	Subject Body Weight (KG)	Drop Height (CM)	Peak GRF (BW)	Ref.
PLF	Military boots	63.2 (5.4)	54	6.2 (1.0)	[4]
PLF	Regulation Army boots	No report	93	9.34	[47]
			137	14.07	
HSPL	Barefoot	57.8 (8.5)	32	16.95 (2.59)	[48]
			52	18.88 (3.46)	
			72	23.98 (4.21)	
HSPL	ES-B	65 (5.2)	80	5.9 (0.38)	Present work
	BS-A			6.98 (1.38)	

## Data Availability

Not applicable.

## Supplementary Materials

The following supporting information can be downloaded at: https://www.mdpi.com/article/10.3390/polym14163270/s1, Figure S1: Unit cell of four typical TPMS topologies; Figure S2: Geometry model of cubic specimens with 4 × 4 × 4 cell numbers for four typical TPMSs; Figure S3: Cubic specimens of TPMS fabricated by SLS technique; Figure S4: Results of quasi-static compression tests of Diamond structure: (a) nominal stress–nominal strain curves; (b) deformation process; Figure S5: Results of quasi-static compression tests of Gyroid structure: (a) nominal stress–nominal strain curves; (b) deformation process; Figure S6: Results of quasi-static compression tests of IWP structure: (a) nominal stress–nominal strain curves; (b) deformation process; Figure S7: Results of quasi-static compression tests of IWP structure: (a) nominal stress–nominal strain curves; (b) deformation process; Figure S8: Fabricated paratrooper boots with the design cushion sole: (a) fabricated paratrooper boots; (b) fabricated paratrooper boots and exoskeletons on lower extremities for jumping.
