# Supplementary material for: A 3D-Printed Sole Design Bioinspired by Cat Paw Pad and Triply Periodic Minimal Surface for Improving Paratrooper Landing Protection
Source: Polymers (Basel). 2022 Aug 11;14(16):3270. doi: 10.3390/polym14163270 (PMC9412508; doi:10.3390/polym14163270)
Supplement: Supplementary file 1 [file polymers-14-03270-s001.zip › polymers-1814789-supplementary.pdf]

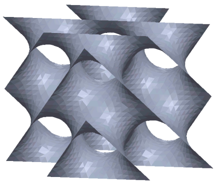

(a) Diamond

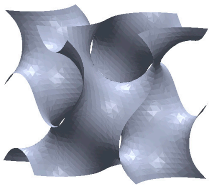

(b) Gyroid

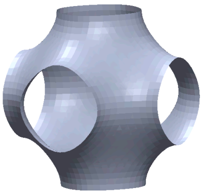

(c) Primitive

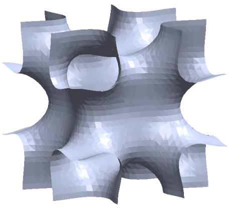

(d) IWP

Figure S1. Unit cell of four typical TPMS topologies.

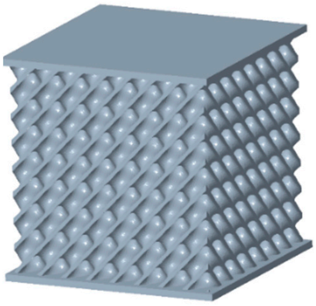

(a) Diamond

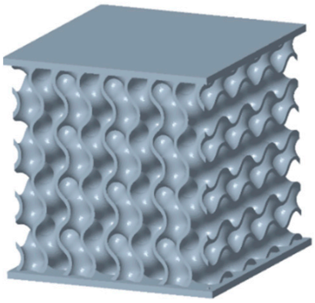

(b) Gyroid

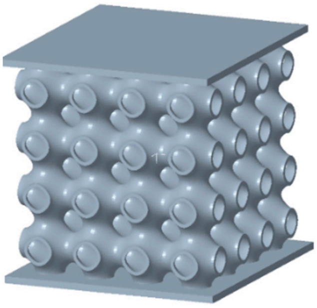

(c) Primitive

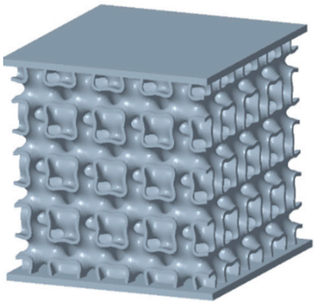

(d) IWP

Figure S2. Geometry model of cubic specimens with 4 x 4 x 4 cell numbers for four typical TPMS.

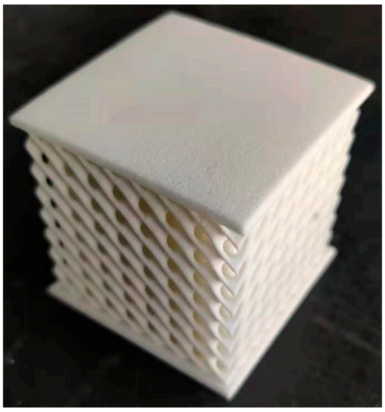

(a) Diamond

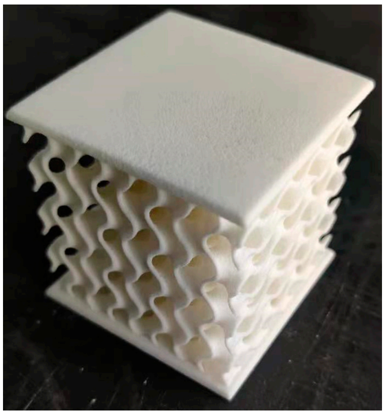

(b) Gyroid

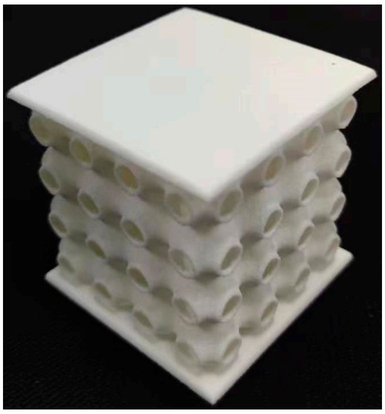

(c) Primitive

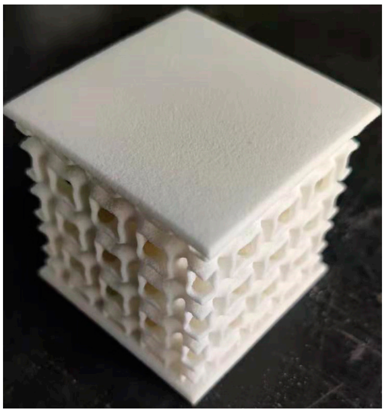

(d) IWP

Figure S3. Cubic specimens of TPMS fabricated by SLS technique**.**

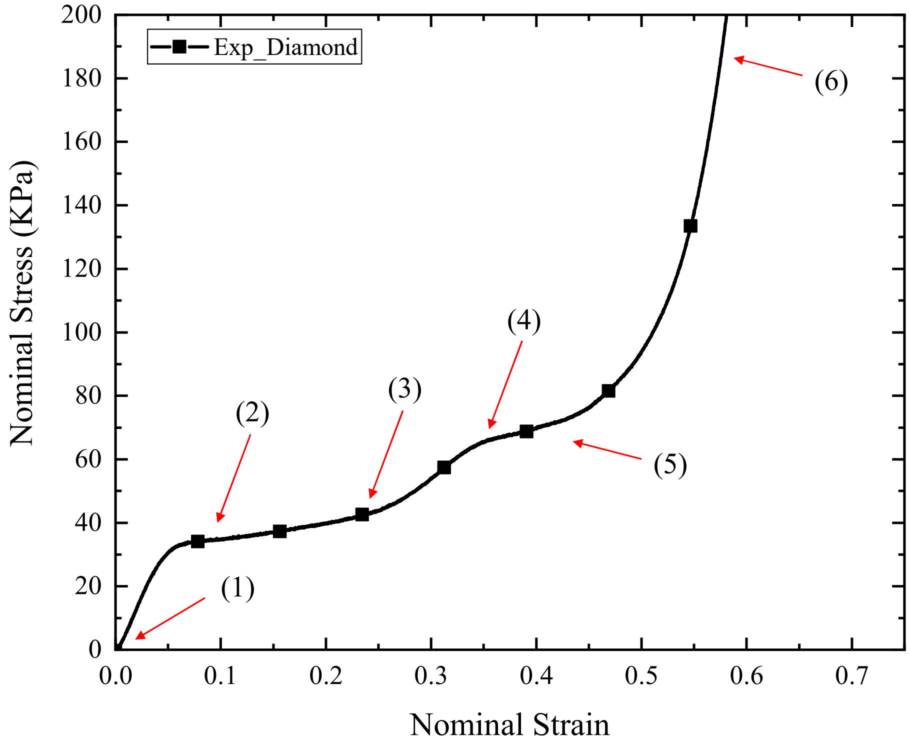

(a)

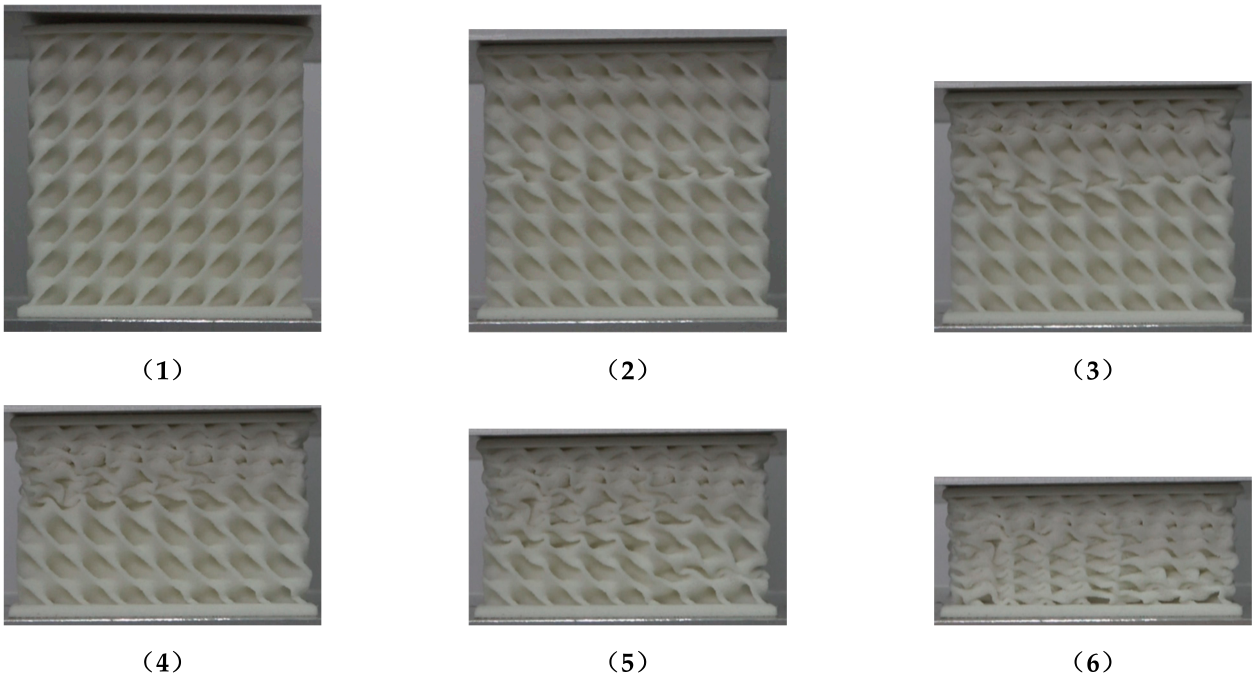

(b)

Figure S4. Results of quasi-static compression tests of Diamond structure: (a) Nominal stress-nominal strain curves; (b) Deformation process.

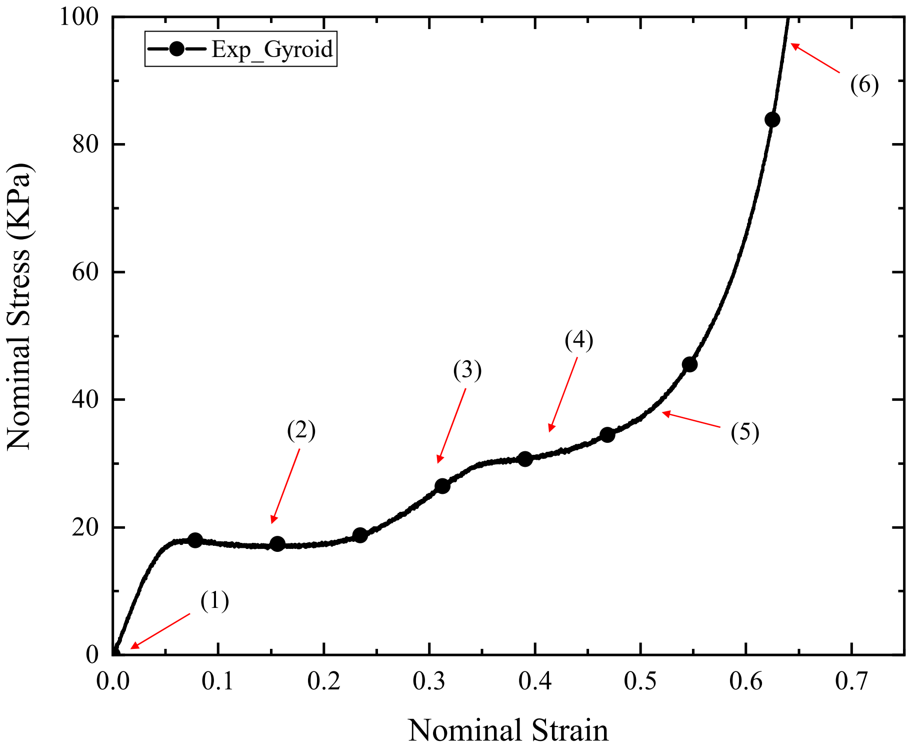

(a)

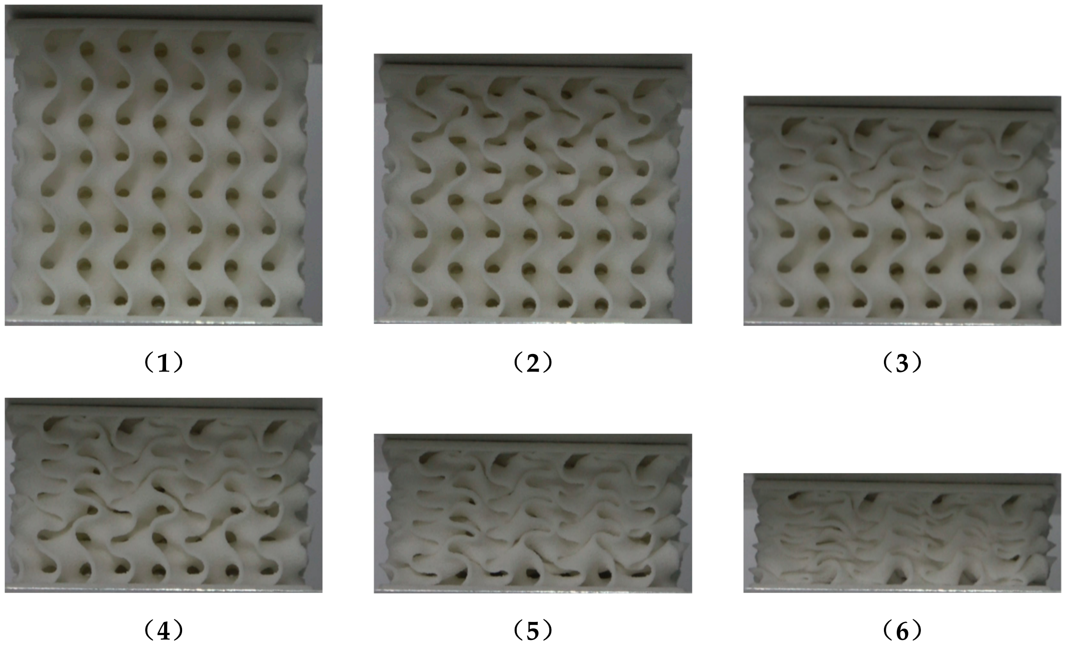

(b)

Figure S5. Results of quasi-static compression tests of Gyroid structure: (a) Nominal stress-nominal strain curves; (b) Deformation process.

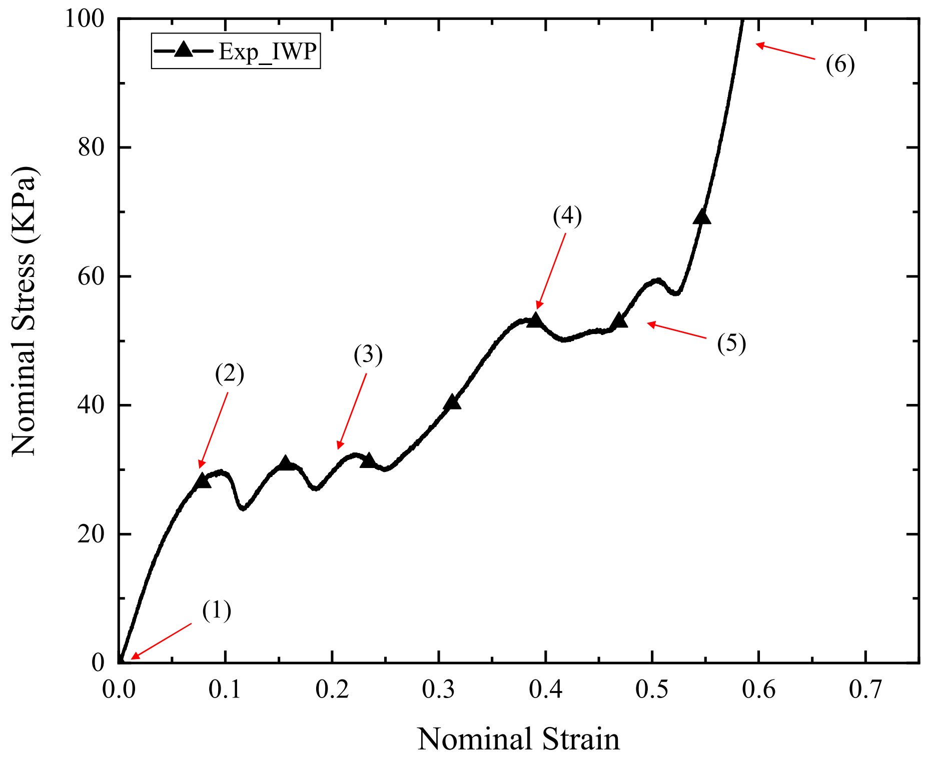

(a)

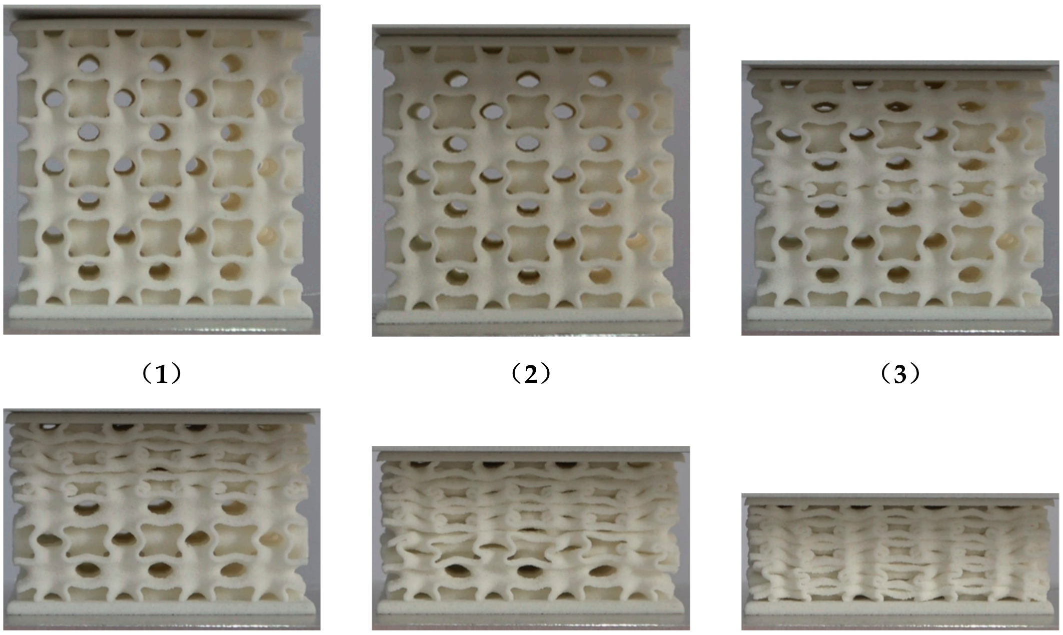

（1） （2） （3）

（4） （5） （6）

(b)

Figure S6. Results of quasi-static compression tests of IWP structure: (a) Nominal stress-nominal strain curves; (b) Deformation process.

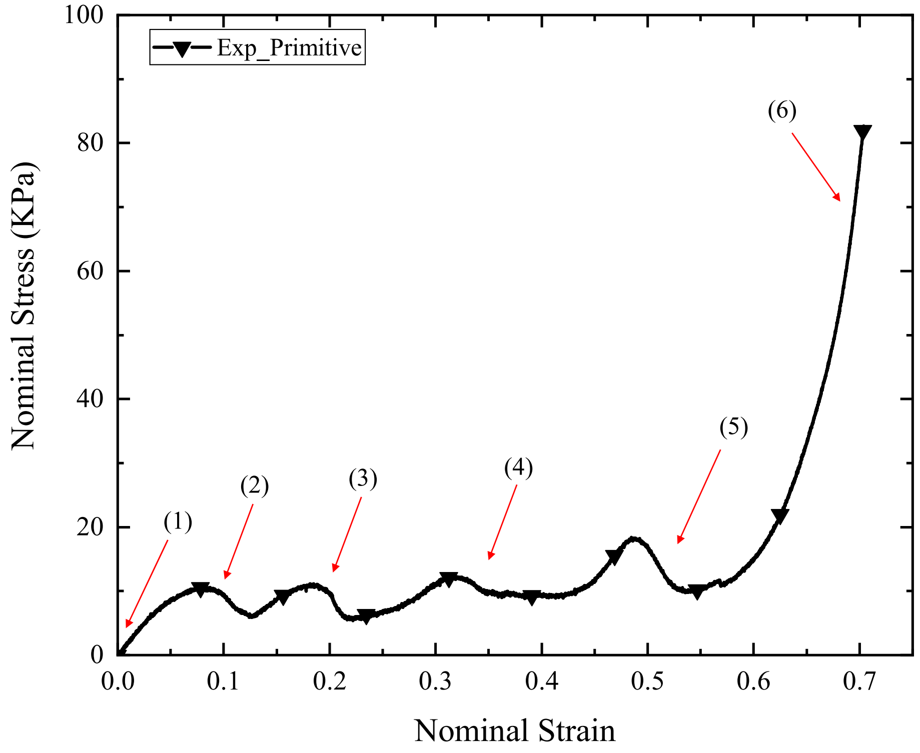

(a)

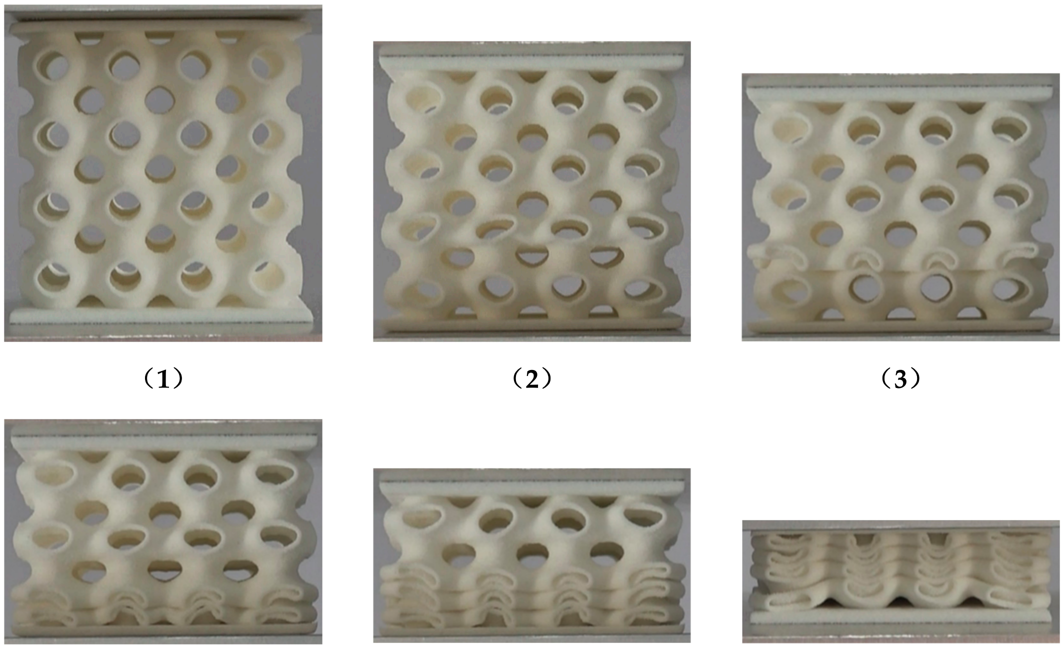

（1） （2） （3）

（4） （5） （6）

(b)

Figure S7. Results of quasi-static compression tests of IWP structure: (a) Nominal stress-nominal strain curves; (b) Deformation process.

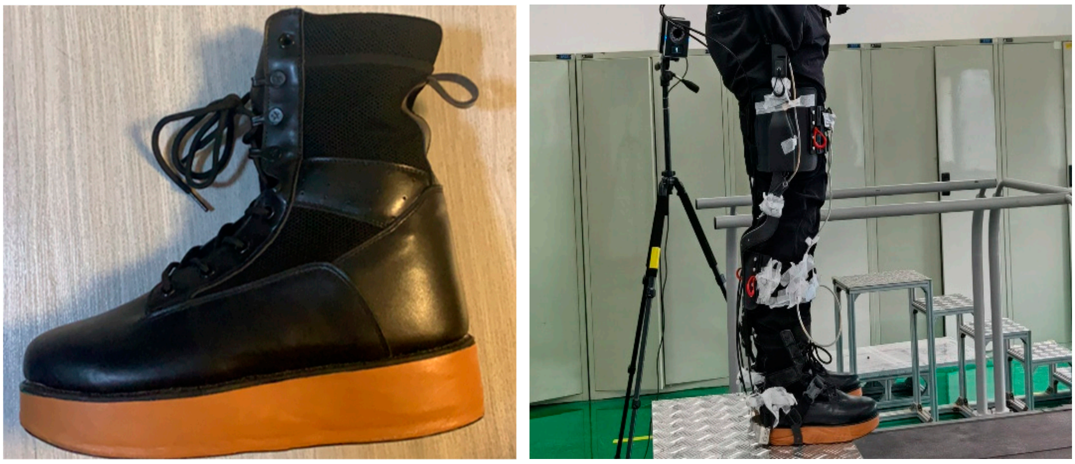

(a) (b)

Figure S8. Fabricated paratrooper boots with the design cushion sole: (a) Fabricated paratrooper boots; (b) Fabricated paratrooper boots and exoskeletons on lower extremities for jumping.
